# A Case of Distal Femur Medial Condyle Hoffa Type II(C) Fracture Treated with Headless Screws

**DOI:** 10.7759/cureus.802

**Published:** 2016-09-23

**Authors:** Chirag Kapoor, Aditya Merh, Malkesh Shah, Paresh Golwala

**Affiliations:** 1 Orthopaedics, Sumandeep Vidyapeeth, Vadodara, Gujarat

**Keywords:** hoffa, herbert screw, posterior approach

## Abstract

Coronal plane fractures of the distal femur are less frequent compared to sagittal plane fractures. They were described by Hoffa in 1904 and are known as Hoffa fractures (AO type B3). They are isolated fractures of the femoral condyle and rare in occurrence. The objective in the treatment of these fractures is to achieve anatomical reduction of the articular surface and a stable fixation to prevent joint damage in future and prevent post-traumatic arthritis of the joint. We report the case of a young male patient who had a rare type of medial Hoffa fracture which was treated by open reduction and internal fixation using headless Herbert screws using a posterior approach. The fracture was united in eight weeks, and the patient had a full range of knee movement. We advocate this approach and modality of treatment for Hoffa type II(C) fractures.

## Introduction

Coronal plane fractures of the distal femur are less frequent compared to sagittal plane fractures and were described by Hoffa in 1904 and are known as Hoffa fractures (AO type B3). They are isolated fractures of the femoral condyle and rare in occurrence [1]. Lateral condyle Hoffa fractures are three times more common than medial condyle fractures [2]. Because of the rarity of medial condyle Hoffa fractures, not much is reported about this injury and its management.

The mechanism of injury has been reported to be a direct anteroposterior force to the flexed and abducted knee for lateral condylar fractures and a direct impact on the medial side of the knee in flexion for a medial condylar fracture. The objective in the treatment of these fractures is to achieve anatomical reduction of the articular surface and a stable fixation to prevent joint damage and post-traumatic arthritis in future [3].

We report the case of a young male patient who had a rare type of medial Hoffa fracture type II(C) which was managed surgically by using a posterior approach. Informed consent was obtained from the subject used in the study.

## Case presentation

A 38-year-old male patient had a road traffic accident and presented to us one day later in the casualty department. He was not able to walk and had pain in the right knee joint posteriorly. There was a tense hemarthrosis of the right knee joint with overlying shiny skin. The knee range of movement was severely restricted due to pain. The patient was vitally stable and had no other associated injury.

**Figure 1:**
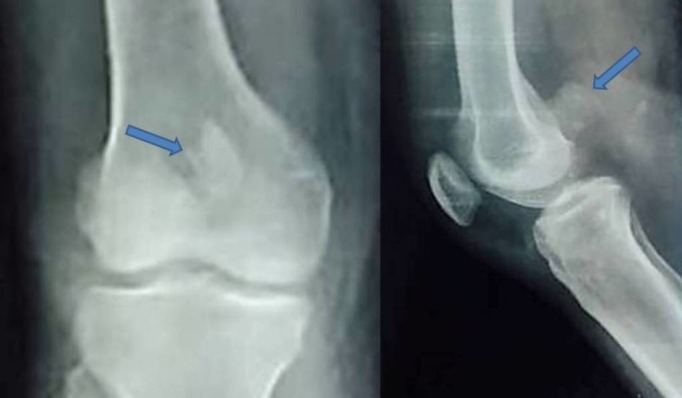
The pre-op Xray images of the anteroposterior & lateral views showing the distal femur medial condyle Hoffa type II(C) fracture

**Figure 2:**
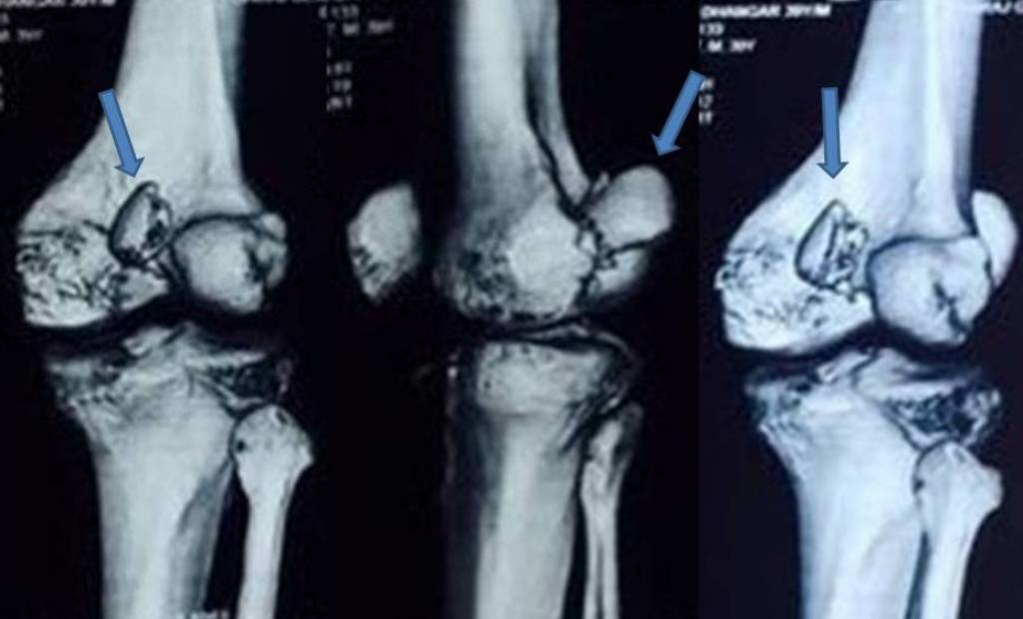
A three-dimensional computed tomography (CT) scan image showing the distal femur medial condyle Hoffa type II(C) fracture

Plain radiographs (anteroposterior, lateral, and oblique views of the right knee joint) showed a small osteochondral fragment of the posterior aspect of medial condyle which was displaced and rotated and was situated just proximal to the intercondylar notch [Figure 1]. A three-dimensional CT scan was done for better visualization of the fracture configuration which confirmed that it was a medial Hoffa type II(C) fracture [Figure 2].

**Figure 3:**
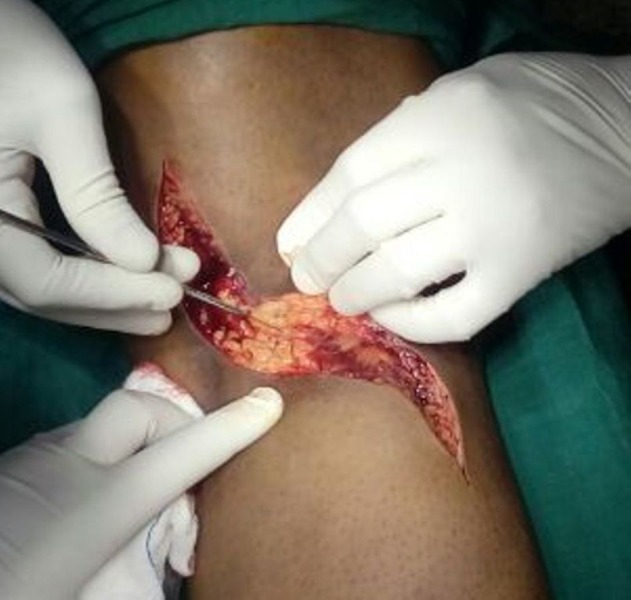
A standard posterior approach to the knee joint using a lazy S-shaped incision

**Figure 4:**
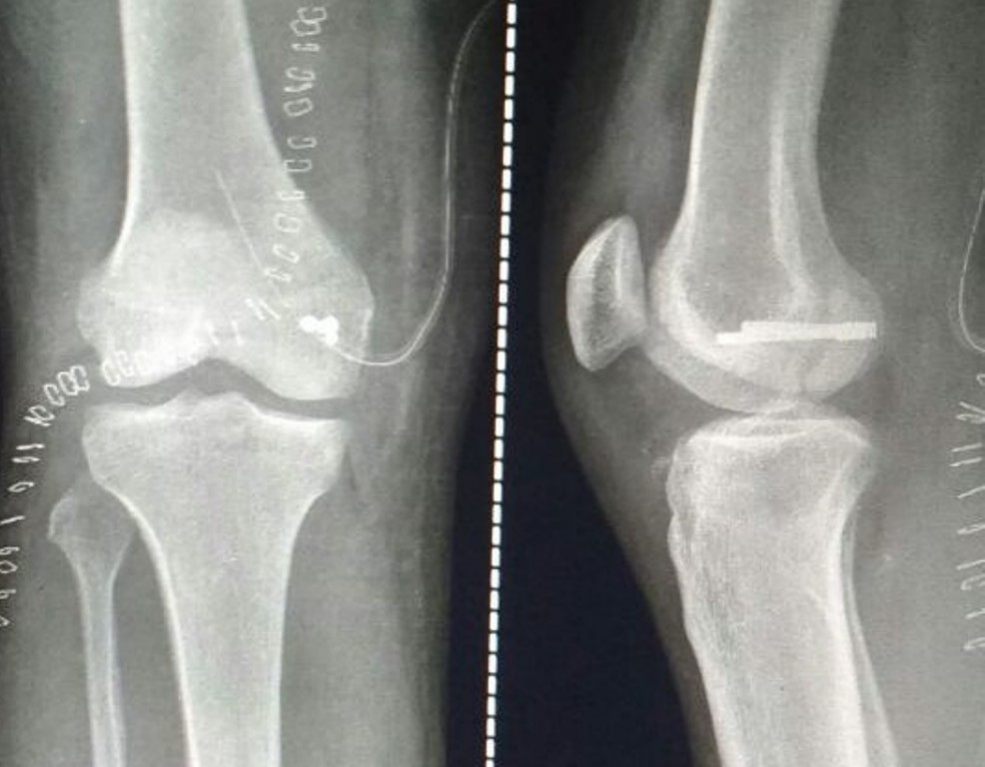
The post-op X-ray images of the anteroposterior and lateral views showing the Hoffa type II(C) fracture fixed with two non-parallel headless Herbert screws after reduction of the fracture.

Initially, the patient was given a cylindrical above-knee slab with limb elevation to reduce knee swelling for three days. Then, after taking a written informed consent from the patient, open reduction and internal fixation with headless screws was done using a direct posterior approach to the knee, with the patient in prone position. A lazy S-shaped incision was used [Figure 3]. The fracture surfaces were cleaned, and the displaced fragments were anatomically reduced and fixed with K-wires. A reduction was done with the knee in 90 degrees of flexion. Once the reduction was done, fixation was carried out first with guide pins. Then two countersink-'Herbert' screws were placed perpendicular to the fracture surface and non-parallel to each other [Figure 4].

**Figure 5:**
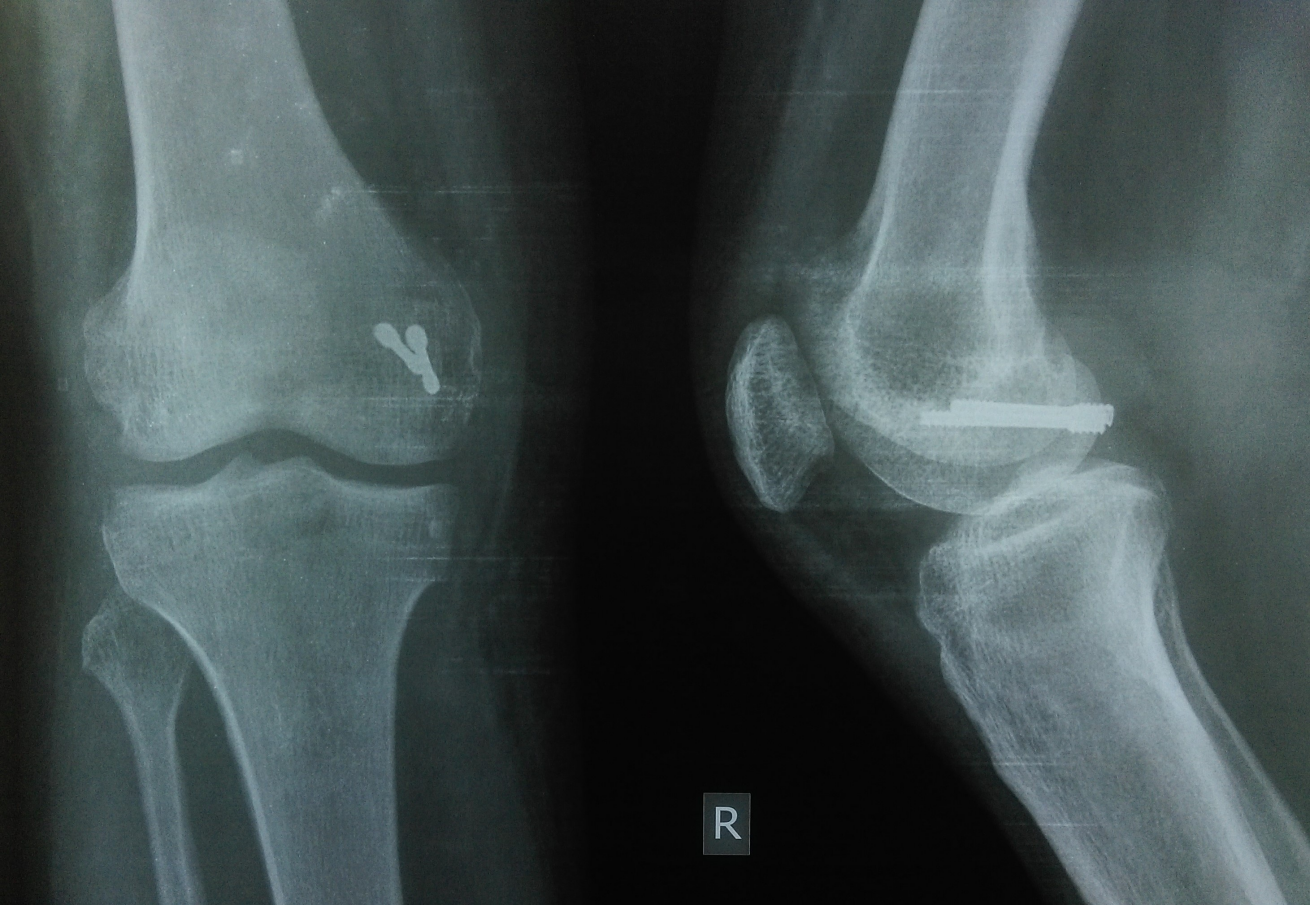
A 10-week follow-up Xray image of the anteroposterior and lateral views showing the union of the fracture with in-situ Herbert screws

The patient was kept non-weight bearing in a cylindrical plaster cast for six weeks. After that, the plaster was removed, and partial weight-bearing was started with a walker for 15 days. At the eight-week follow-up, the X-ray showed a union of the fracture [Figure 5] and the patient was made to walk full weight-bearing without support. The knee joint flexion and extension exercises were started, and zero degrees to 125 degrees range of movement was achieved by ten weeks postoperatively.

## Discussion

Intra-articular coronal plane “Hoffa” fractures of the distal femur medial condyle are rare injuries and difficult to treat. Conservative management often leads to unsatisfactory results and nonunion as many undisplaced fractures get displaced after conservative treatment. This makes the prognosis of such injuries worse.

If the fragment is not reduced properly, roughening of the articular surface and osteonecrosis occur, which may produce a marked disability in future. Moreover, if the fragment is small, it should not be removed because it is an important part of the articular surface when the knee is flexed at 90 degrees. So, it is mandatory to fix this fragment anatomically to achieve a good outcome.

**Figure 6:**
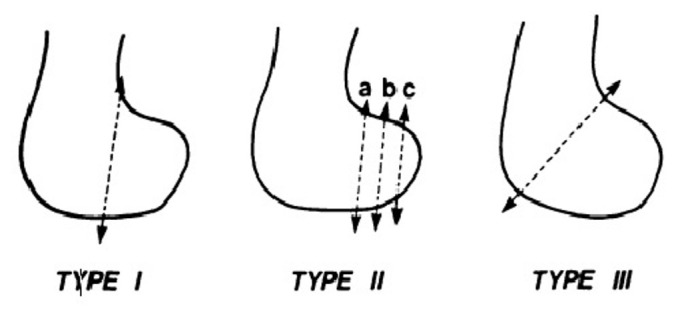
Classification of Hoffa fracture by Lewis et al.[4] Classification of Hoffa fracture by Lewis et al. [4]

Lewis, et al. [4] classified the Hoffa fractures into three types. In Type I and Type III Hoffa fractures there are some soft tissue elements attached to the fractured condylar fragment to provide blood supply to this fragment. But in Type II Hoffa fractures there is no soft tissue element attached to the fractured condylar fragment [Figure 6].

Up to 30% of these fractures get overlooked on plain radiographs and require computerized tomography to assess the fracture pattern and plan the surgical fixation [5]. We also preferred to get a three-dimensional CT done so as to assess the fracture configuration properly.

It is accepted that screw fixation is a good fixation method for treating Hoffa fractures. There is no standardized surgical approach in treating these fractures, and usually, an anterolateral or anteromedial incision is used (depending on the condyle involved) for Type I and III fractures and two anteroposteriorly placed lag screws are inserted from the non-articular area just proximal to the patella-femoral joint to engage the fractured condylar fragment. In Type II Hoffa fractures, because the fracture line is near the articular cartilage of the posterior condyle, a posterior approach and two postero-anteriorly placed lag screws are preferred [6, 7]. However, the screws are inserted through the articular surface, so the screw heads should be countersunk or headless Herbert screws should be used instead of compression screws.

In our case it was a Type II(C) fracture, so we preferred a posterior approach as the fragment was too small to get held by anteroposterior screws, was rotated and displaced posterior to the intercondylar notch, and was difficult to reduce using the anteromedial approach. So we preferred to approach the fracture posteriorly and fix it with posteroanterior Herbert screws in a non-parallel fashion rather than anteroposterior screws. Lag screws placed in a posteroanterior direction are more stable than anteroposteriorly placed lag screws [8] and crossed screws are more rigid than the parallel screws [9].

## Conclusions

We believe that these rare injuries should be identified in time and treated aggressively by an early open reduction and an anatomically rigid internal fixation, to achieve a good recovery of the function of the joint.
